# Promoting sunscreen use and skin self-examination to improve early detection and prevent skin cancer: quasi-experimental trial of an adolescent psycho-educational intervention

**DOI:** 10.1186/s12889-018-5570-y

**Published:** 2018-05-29

**Authors:** Gill Hubbard, Richard G. Kyle, Richard D. Neal, Vincent Marmara, Ziyan Wang, Stephan U. Dombrowski

**Affiliations:** 1 School of Health, Social Care and Life Sciences, Centre for Health Sciences, University of the Highlands and Islands (UHI), Old Perth Road, Inverness, IV2 3JH Scotland; 2 School of Health and Social Care, Edinburgh Napier University, Sighthill Court, Edinburgh, EH11 4BN UK; 3 Academic Unit of Primary Care, Institute of Health Sciences, University of Leeds, Worsley Building, Leeds, LS2 9NL UK; 4 Department of Management, Faculty of Economics, Management and Accountancy, University of Malta, Humanities B (FEMA), Msida, MSD 2080 Malta; 5 Division of Psychology, Faculty of Natural Sciences, University of Stirling, FK10 4LA Stirling, Scotland

**Keywords:** Skin cancer, Skin self-examination, Adolescence

## Abstract

**Background:**

Skin cancer rates are increasing. Interventions to increase adolescent sunscreen use and skin self-examination (SSE) are required.

**Methods:**

Quasi-experimental design; 1 control and 4 intervention group schools in Scotland, UK. Participants were 15–16 year old students on the school register. The intervention was a theoretically-informed (Common-Sense Model and Health Action Process Approach) 50-min presentation, delivered by a skin cancer specialist nurse and young adult skin cancer survivor, to students in a classroom, supplemented by a home-based assignment. Outcome variables were sunscreen use intention, SSE intention/behaviour, planning, illness perceptions and skin cancer communication behaviour, measured 2 weeks pre- and 4 weeks post- intervention using self-completed pen and paper survey. School attendance records were used to record intervention up-take; students self-reported completion of the home-based assignment. Pearson’s chi-square test, analysis of variance, and non-parametric Wilcoxon Signed Ranks Test were used to measure outcomes and associations between variables. Focus groups elicited students’ (*n* = 29) views on the intervention. Qualitative data were analysed thematically.

**Results:**

Five of 37 invited schools participated. 639 (81%) students in intervention schools received the intervention; 33.8% completed the home-based assignment. 627 (69.6%) of students on the school register in intervention and control schools completed a questionnaire at baseline; data for 455 (72.6%) students were available at baseline and follow-up. Focus groups identified four themes – personal experiences of skin cancer, distaste for sunscreen, relevance of SSE in adolescence, and skin cancer conversations. Statistically significant (*p* < 0.05) changes were observed for sunscreen use, SSE, planning, and talk about skin cancer in intervention schools but not the control. Significant associations were found between sunscreen use, planning and 2 illness perceptions (identity and consequence) and between SSE, planning and 3 illness perceptions (timeline, causes, control).

**Conclusions:**

It is feasible to promote sunscreen use and SSE in the context of an adolescent school-based psychoeducation intention. Further research is required to improve study uptake, intervention adherence and effectiveness.

**Trial registration:**

ISRCTN11141528

**Electronic supplementary material:**

The online version of this article (10.1186/s12889-018-5570-y) contains supplementary material, which is available to authorized users.

## Background

### Skin cancer prevention

The burden of skin cancer in Europe is high and increasing rapidly [1]. Skin cancer incidence has increased in the United Kingdom (UK) by 360% since the 1970s [2], with an estimated 86% of these cases attributable to excessive sunlight exposure [3]. Sunburn is implicated in the pathogenesis of different skin cancers including squamous cell carcinoma [4], basal cell carcinoma [5], and melanoma [6]. While rare, the recent rapid increase in skin cancer incidence in adolescence is attributed to environmental (e.g., excessive sun exposure leading to sunburn) and genetic factors [7–10]. Sunburn in childhood and adolescence heightens the risk of skin cancer in adulthood [6, 11–14]. For example, one United States cohort study found that the overall lifetime risk of developing skin cancer nearly doubled (OR = 1.80 (95% CI, 1.42–2.28) if participants experienced five blistering sunburns between the ages 15 and 20 years [14]. Sun protection strategies (e.g., sunscreen use, covering up with suitable clothing, avoiding going out in the sun) act by blocking or diminishing the contact of ultraviolet radiation (UV) with the skin, thus, avoiding DNA damage and the development of skin cancer [15]. A major concern is poor sunscreen application in this age group [16–21]. In one study, adolescents were found to deliberately use a sunscreen with a low sun protection factor and delay application of sunscreen to get a tan [20]. Hence, specific instructions on the correct application and re-application of sunscreen and use of other sun protection behaviours during adolescence are urgently needed [22]. There are other reasons why adolescents are a target group for sun safety interventions. Multiple risk behaviours – smoking, hazardous alcohol consumption, low levels of physical activity, poor diet and excessive sun exposure – cluster in adolescence [23]. Many health problems manifested in adulthood have antecedents in childhood [24–26]. Behaviours (e.g. sunbathing) and attitudes (e.g. pro-tanning) associated with skin cancer emerge in adolescence and track into adulthood [27, 28]. Adolescence therefore provides a critical window of opportunity for the primary prevention of skin cancer caused by sunburn across the life-course.

### Early detection of melanoma

There is no UK guidance for how often an individual should examine their skin but there is a recognition to make an appointment with a doctor if an individual notices a change to a mole, freckle or normal patch of skin [29]. The International Skin Cancer Foundation recommends a head-to-toe self-examination every month [30]. Early detection of melanoma improves survival, disease- and treatment-related morbidity and psychological adjustment [31, 32]. A recent systematic review of eight studies showed that shorter onset to melanoma diagnosis is associated with more favourable clinical outcomes [33]. A landmark case-control study found that skin self-examination (SSE) may decrease mortality from melanoma by 63% [34], although there is lack of sufficient evidence to know with certainty the effect of SSE on melanoma incidence and stage at presentation [35]. Nonetheless, evidence suggests that over half of all melanomas are self-detected [35–37]. Learning to conduct SSE during adolescence may increase the chances of this important health behaviour becoming habitual [38] during adulthood when skin cancer incidence is higher. One study found that self-detection is associated with knowledge of the ABCD (asymmetry, border, colour, diameter) criteria used to examine moles and performance of SSE [39]. It is concerning, therefore, that a recent UK study found that less than half of adolescents recognized ‘change in the appearance of existing or new mole’ as a melanoma warning sign [16] and another study found that SSE in young people is rare [40]. Moreover, few young people talk about cancer [41]; yet, advice from family and friends is a trigger to seeking professional advice about a symptom suggestive of cancer [42].

### Behaviour change interventions

Interventions that simultaneously promote sunscreen use to prevent skin cancer and SSE to improve early detection may be effective. A recent mobile phone text messaging intervention, targeting either sun protection or SSE in a population aged 18 to 42 years at high risk of skin cancer, found that the SSE group improved their sun protection behaviours to a similar degree as that observed in the sun protection group [43]. It concluded that repeated messages about early detection of skin cancers and encouragement to conduct SSE prompt people to consider skin cancer risk more broadly.

A review of educational interventions published in 2004 concluded that simply raising awareness of the risks of UV radiation may go some way towards improving sun protection behaviours during adolescence but is unlikely to directly translate into sustained behaviour change on its own [44]. More recent studies have therefore focused on a range of behavioural determinants, acknowledging that individuals must be motivated to perform specific behaviours (e.g., sunscreen use and SSE) and motivation must be directly translated into actual behaviours [45].

Risk perceptions are social cognitions identified by many behavioural theories as primary motivators of a range of health behaviours [46]. Recent meta-analytic evidence suggests that interventions that successfully engage and change risk perceptions produce subsequent improvements in health behaviours [47]. This proposition is supported by empirical evidence of associations between risk perceptions and sun protection behaviours in adolescents [18, 21, 48, 49] and SSE in adults [50, 51]. Illness representations are another class of social cognitions that determine health behaviours and refer to an individual’s beliefs and expectations about an illness [52, 53]. Illness representations are central to the Common-Sense Model of illness representation and self-regulation (CSM); a study in young adults found that CSM dimensions *identity* (e.g., ‘I have white or pale skin’), *cause* (e.g., ‘Lots of sun exposure to skin without using sunscreen’) and *timeline* (e.g., ‘People of my age are likely to develop skin cancer’) were associated with higher SSE intentions [54]. Expanded versions of CSM such as the Cognitive-Social Health Information Processing (C-SHIP) model include risk perceptions [55]. Illness perceptions (the term we use that includes risk perceptions and illness representations) are multi-dimensional and in the context of this study include adolescents’ beliefs about the causes of skin cancer and their perceived ability, confidence and relevance to control for skin cancer risk at their age.

According to the Health Action Process Approach [45], behavioural intentions are more likely to be translated into action when people generate specific plans [56]. In the context of sunscreen use, empirical evidence suggests that adolescent frequent sunscreen users are more likely to use action plans related to sunscreen use (e.g., by planning to take sunscreen with them to use when at the pool or beach, during sports and when engaging in outdoor activities) compared to infrequent sunscreen users [18]. Indeed, the study found that use of action plans was the strongest predictor of sunscreen use [18].

### Study aims

The intervention being tested in this feasibility study was designed to improve sun protection behaviours to prevent skin cancer and SSE to improve early detection of skin cancer. This feasibility study was for preparation of a future effectiveness trial of an intervention to increase sunscreen use and SSE during adolescence and received ethical approval from the University of Stirling Research and Ethics committee (SREC 15/16 – Paper No.66 – Version 2). The following trial parameters were measured to assess the feasibility of trial procedures: number of schools agreeing to participate, adolescent consent rate, and intervention adherence and acceptability. We hypothesised that we would observe at baseline associations between: a) sunscreen use intention and sunscreen use planning; b) SSE behaviour and SSE planning; c) SSE intention and SSE planning; d) SSE behaviour and illness perceptions; e) SSE intention and illness perceptions; f) sunscreen use intention and illness perceptions. A further purpose was to determine if a psycho-educational intervention (‘Do you know your skin?’) worked as intended and that we would observe the following changes in outcomes from baseline to follow-up in adolescents’: i) sunscreen use intention and sunscreen use planning; ii) SSE behaviour, SSE intention and SSE planning; iii) skin cancer perceptions; and iv) talk about skin cancer in intervention group schools but not the control group schools.

## Methods

### Design

The feasibility study used a quasi-experimental design, with four schools allocated by the research team to an intervention group one to a control group, to find out if the intervention worked as intended and examine trial procedures. This was not a full randomised controlled trial; the study is not powered to measure effectiveness and there was no aim to recruit a representative sample.

### Study population and recruitment

The criteria for inclusion in the study were: males and females aged 15–16 years of age. Participants were recruited from five secondary schools in Scotland. Four schools were allocated to the intervention group and one to a control group (which received the intervention after the study). A letter was sent to 35 state and independent secondary school head teachers in one city in Scotland inviting participation, which was followed up by telephone and/or email to arrange a face-to-face meeting to discuss the study and obtain agreement for the school’s participation. Due to poor response, two schools in a different area in Scotland, that were already known to the research team through previous work, were invited to participate.

Once a school had consented to participate, the named parent/carer on the school register was sent a study information pack, which included a form to be returned to the school if they wished to opt their child out of the study. The parent/carer was given the opportunity to contact the research team to discuss the study by telephone or email. Students who were not opted out by a parent/carer were given their study information sheet and consent form in the classroom. Students who had been opted out of the study were given education assignments to do while their classmates completed the questionnaires.

### Intervention description

The intervention was developed by the research team with the support of an expert working group that included two people who had been treated for skin cancer, three experts in health behaviour change, one policy-maker in cancer early detection, one skin cancer specialist nurse and one dermatologist. The intervention had two parts: a presentation delivered to students in school and a home-based assignment.

A practicing skin cancer nurse specialist delivered a 50-min presentation to students about skin cancer and SSE. Each presentation was delivered by the nurse on one occasion during the school day in a classroom. After playing a 5-min video ‘Dear 16-year-old me’ (http://dcmf.ca), the nurse delivered the presentation with the aid of Microsoft PowerPoint slides and covered: personal experiences of skin cancer, incidence patterns, risk factors, associations between disease staging and survival, and benefits of SSE. A young adult skin cancer survivor gave a brief 5-min talk after the nurse-delivered presentation. The talk was about his personal experience of melanoma diagnosis at 16 years old, impacts on his life and his views on sunscreen use and SSE behaviour.

A home-based assignment comprised a booklet with instructions. Adolescents were given an exercise to self-examine their skin and asked to complete an action plan for regular monthly sunscreen use and an action plan for SSE. The SSE component of the booklet had three sections: a) information on the importance of planning; b) instructions of what should be included in the plan; c) formulating ‘if-then’ action plans (e.g., If I am having a shower then I will check my skin) and coping plans (e.g. To make sure I don’t forget, I will add the appointment to my calendar and put a reminder post-it on the fridge).

The school allocated to the control group, received the same intervention after the study had ended.

### Intervention adherence and acceptability

We assessed intervention adherence and acceptability both objectively and via self-report. Intervention adherence was defined in two ways: proportion of eligible adolescents on a school register receiving the presentation, and number of participating adolescents completing the home-based assignment. The number of adolescents in intervention schools who received the presentation was objectively measured using school attendance records. The number of adolescents doing the home-based assignment was self-reported at follow- up.

Focus groups were conducted in intervention group schools to explore adolescents’ opinions about relevance, content, format and delivery methods. Focus groups (*n* = 3; 1 intervention group school was unavailable due to exam revision timetabling) to elicit adolescents’ views on the intervention’s relevance, content, format and delivery methods were conducted approximately 8 weeks after the intervention. Focus groups were audio-recorded and took place during school time, in a classroom, at a time and place selected by the teacher and lasted approximately 50 min. Confidentiality was explained and informed consent was obtained in writing.

### Variables and measures

Outcome variables were measured 2 weeks before (baseline) and 4 weeks after (follow-up) the intervention in intervention schools using self-completed pen and paper survey. Teachers administered the questionnaires in the classroom. Parallel time-points were used in the control school. Only students who had consented to participate in the study completed questionnaires at these time-points. We adapted items from action and coping planning scales [57] to measure sunscreen planning and SSE planning. Items to measure illness perception were adapted from a study about SSE in young adults [54], that in turn had used items used in the Illness Perception Questionnaire [58].

#### Sunscreen use intention

Sunscreen use intention was measured using one item: ‘Do you intend to use a high factor sunscreen if you are going out in the sun?’ A five-point continuous rating scale was used to gauge response from 1 (definitely will not) to 5 (definitely will). We did not measure sunscreen use behaviour because the feasibility study was conducted in winter (January – March) and not during the summer months when sunscreen use would be highly relevant in Scotland.

#### Sunscreen use planning

Sunscreen use planning was measured using five items: ‘I have made a detailed plan on… i) *What* sunscreen I will use; ii) *When* I will use it; iii) *Where* I will get it from; iv) *How* I will remember to carry it’; and, iv) *What* to do if I am tempted not to use it.’ A five-point continuous rating scale was used to gauge response from 1 (completely disagree) to 5 (completely agree).

#### SSE behaviour and intention

SSE behaviour was measured using one item: ‘In the past month, have you examined your skin for signs of possible skin cancer?’ Responses were yes, no or don’t know. SSE intention was measured using one item: ‘Do you intend to examine your skin for signs of possible skin cancer on a regular basis.’ A five-point continuous rating scale was used to gauge response from 1 (definitely will not) to 5 (definitely will).

#### SSE planning

SSE planning was measured using four items: ‘I have made a detailed plan regarding… i) *When* to examine my skin for signs of possible skin cancer; ii) *Where* to examine my skin for signs of possible skin cancer; iii) *How* to examine my skin for signs of possible skin cancer’; and, iv) ‘I have made a plan for dealing with things that could stop me from examining my skin for signs of possible skin cancer.’ A five-point continuous rating scale was used to gauge response from 1 (completely disagree) to 5 (completely agree).

#### Talking about skin cancer

Talking about skin cancer was measured using one item: ‘Have you spoken to anyone about skin cancer in the last month?’ Responses were yes or no.

#### Illness perceptions

Five dimensions of the CSM were measured using the following items:

- *Identity*: ‘How much would getting skin cancer affect your life?’ An eleven-point continuous rating scale was used to gauge response from 0 (not at all) to 10 (it would severely affect my life);
- *Control:* ‘How much control do you feel you have to prevent yourself from getting skin cancer? An eleven-point continuous rating scale was used to gauge response from 0 (not at all relevant) to 10 (very relevant).
- *Timeline*: ‘Given your age, how relevant is it to regularly examine your skin for signs of possible skin cancer?’ An eleven-point continuous rating scale was used to gauge response from 0 (not at all relevant) to 10 (very relevant).
- *Consequence*: ‘How painful do you think the effects of treatments for skin cancer would be? An eleven-point continuous rating scale was used to gauge response from 0 (not at all painful) to 10 (extremely painful).

The following open-ended question was used to measure the CSM dimension *cause*: ‘Please list in rank-order the three most important factors that you believe cause skin cancer.

#### Social-demographic characteristics

Socio-demographic questions were included to gather data on sex and ethnicity. Using UK government statistical service guidance [59], students were asked to choose from a list of 5 categories (White, Mixed, Asian or Asian British, Black or black British, Chinese/other) which best describes their ethnic group.

### Analyses

Quantitative data analysis was conducted in three steps. First, descriptive statistics were calculated for sociodemographic variables (i.e., age, gender) and outcome variables (e.g., sunscreen use intention and planning, SSE intention, behaviour and planning, skin cancer illness perceptions, and talking about cancer) at baseline and reported as n (%) for categorical data and mean (Standard Deviation [SD]) for continuous data. As described above, one illness perception dimension – *causes -* was measured using an open-ended question. We drew on the widely cited Dahlgren and Whitehead’s [60] rainbow model of the main determinants of health as a framework to help identify the broad range of factors that adolescents’ perceived to cause skin cancer. The open answers were categorised into, 1) age, sex and constitutional factors (e.g., age; genetics; moles; skin type; random mutation; family history; hereditary), 2) individual lifestyle factors (e.g. diet; lack of sun protection; lack of skin examination; sunburn; lack of skincare; sunbeds; tanning beds; lack of hygiene; tattoos), and 3) general environmental conditions (e.g. sun; sun exposure; radiation; UV light; pollution; heat). Second, Pearson’s chi-square test was used to assess associations between sunscreen use intention/SSE intention and behaviour, and planning behaviour at baseline. Analysis of Variance (ANOVA) was used to assess associations between SSE behaviour, intention and each of the 5 CSM dimensions at baseline. Third, to assess change in outcome measures between baseline and follow-up the non-parametric Wilcoxon Signed Ranks Test was used because data were not normally distributed. The test for significance was a within-group comparison (e.g. difference between baseline and follow-up scores within intervention group schools). The study was not designed or powered to definitively measure effectiveness. However, to assess the potential influence of confounders on results sensitivity analyses were conducted. There was no statistically significant difference between the intervention and control groups in terms of gender or having a close family member with cancer, but there was a statistically significant difference of 1.2 years in the mean age of the two groups. Repeated measures ANCOVA and logistic regression were used to adjust for age in outcome analyses. Data were analysed using SPSS Statistics v21 (IBM Corporation, Armonk, NY). Significance tests were two-sided; *p* < 0.05 was considered statistically significant.

Audio-recorded qualitative data from focus groups were transcribed verbatim and analysed thematically using the Framework approach [61]. Qualitative findings provided contextual and explanatory understandings of adolescents’ experiences of the intervention.

## Results

### Sample characteristics

No parent/carer opted their child out from the study and no student declined to participate in the study. According to school records, there were 901 eligible students. 627 (69.6%) completed a questionnaire at baseline. Student absence from school during examination revision period explains why we did not achieve a 100% response rate. A CONSORT pilot and feasibility flowchart is available in a Additional file 1.

The sample included 627 (female: 45.1%, *n* = 283) adolescents with a mean age of 16.1 years (SD = 0.874); 88.0% (*n* = 552) were ‘White’ ethnic background; 10.7% (*n* = 67) of adolescents had a close family member who had skin cancer. Complete data were available for 79 (61%) and 376 (48%) adolescents in the control and intervention schools respectively (i.e., we could pair baseline and follow-up for 455 students for analysis of change in outcome measures).

### Intervention adherence

 Eighty-three percent  (*n* = 639) of adolescents in four intervention group schools (*n* = 771) received the presentation (85% *n* = 150; 75% *n* = 60; 78% *n* = 132; 87% *n* = 297 in each school respectively). This is higher than the total number of students in all five schools completing baseline and/or follow-up questionnaires and indicative of the difference in student absence from school on days when the intervention was delivered and questionnaires administered in schools. 33.8% (*n* = 148) of adolescents in intervention schools reported that they did the home-based assignment to conduct skin self-examination; 65.5% (*n* = 287) reported that they did not do the home-based assignment.

### Intervention acceptability

Focus group interviews in three intervention schools with 29 students (6, 10 and 13 participants in each school, respectively) were conducted. It was not feasible to conduct a focus group in one intervention school due to timetabling constraints during the period when students were preparing for exams. The following key themes were identified – personal experiences of skin cancer, distaste for sunscreen, relevance of SSE in adolescence and skin cancer conversations. Quotations to illustrate each theme are presented in a Additional file 2.

Two components of the presentation focused on *personal experiences of cancer*: the ‘Dear 16 year old me’ film that was shown at the beginning of the presentation and the brief talk from the young adult skin cancer survivor at the very end of the presentation delivered by the skin cancer specialist nurse. Adolescents could recall these personal stories because they were ‘real’, ‘emotional’ and they could ‘relate’. Many adolescents said that the talk given by the young adult cancer survivor was the best feature of the presentation. Nonetheless, some adolescents found the ‘Dear 16 year old me’ film too contrived and ‘patronising’ because it was trying to be too ‘cool’ and ‘down with the kids’. Adolescents could recall a key presentation message i.e., sunburn increases the risk of skin cancer. Nonetheless, many adolescents expressed *distaste for sunscreen* because of its smell and texture (e.g. thickness, greasiness) and put them off using it. Thus, while the intervention appeared to improve awareness about the health risks of excessive sun exposure, this may not be sufficient to increase sunscreen use. Adolescents could recall a key presentation message i.e., to regularly conduct SSE. Some adolescents reported that they very quickly examined their skin after the presentation. Some adolescents did not do the home-based assignment because in contrast to other homework tasks, there was no date when it had to be completed by. Nonetheless, even were the home-based assignment made compulsory, SSE is unlikely to be sustained. This is because adolescents questioned the *relevance of SSE in adolescence*. They understood from the presentation that skin cancer prevalence was higher in adulthood and therefore did not see the relevance of conducting SSE during adolescence. Making SSE habitual by starting the behaviour during adolescence may therefore prove difficult to instigate. Some adolescents reported that they mentioned very briefly to a parent/carer about the presentation that they had about skin cancer. Others gave the impression that when they mentioned the presentation it provided the parent with an opportunity to reinforce key messages about sun protection. Some adolescents reported that their parent/carer looked at the home-based assignment booklet or they spoke about skin cancer with a parent/carer following the presentation. This suggests that school-delivered interventions with a home-based assignment component may reach a wider audience (e.g., parents/carers) than the direct target group (e.g., adolescents).

### Baseline associations

#### Sunscreen use intention, planning and risk perceptions

At baseline, 30.9% (*n* = 191) of adolescents definitely intended to use a high factor sunscreen if they were going out in the sun, and 5.3% (*n* = 33) definitely did not intend to use a high factor sunscreen (Table 1). The difference between male and female intentions were statistically significant; more females definitely intended to use sunscreen than males (37.5% (*n* = 105) versus 25.4% (*n* = 86), *p* < 0.001) (Table 1).

**Table 1:** Sunscreen Use and Skin Self Examination (SSE) intention at baseline by gender

		Total		Male		Female		
Intention		%	*n*	%	*n*	%	*n*	Sig.
Sunscreen Use	1 - Definitely will not	5.3	(33)	6.8	(23)	3.6	(10)	0.001**
	2	11.0	(68)	14.2	(48)	7.1	(20)	
	3	25.2	(156)	26.8	(91)	23.2	(65)	
	4	27.6	(171)	26.8	(96)	28.6	(80)	
	5 – Definitely will	30.9	(191)	25.4	(86)	37.5	(105)	*p*<0.001***
SSE	1 - Definitely will not	13.3	(83)	17	(58)	8.8	(25)	0.041*
	2	29.5	(184)	29.3	(100)	29.7	(84)	
	3	41.8	(261)	38.4	(131)	45.9	(130)	
	4	10.7	(67)	10.9	(37)	10.6	(67)	
	5 – Definitely will	4.6	(29)	4.4	(15)	4.9	(14)	
Have you spoken to anyone about skin cancer in the last month?	Yes No	9.2 90.8	(57) (563)	8.2 91.8	(28) (312)	10.4 89.6	(29) (251)	0.363

Note: *p < 0.05, **p < 0.01, ***p < 0.001

14% (*n* = 87) of adolescents had made a detailed plan W*hen* to use sunscreen; 10.8% (*n* = 67) *What* sunscreen to use*;* 16.7% (*n* = 104) *Where* to get sunscreen from; 8.8% (*n* = 55) *How* to get sunscreen (23.8%, *n* = 148); and 8.7% (*n* = 54) had made a *Coping plan* for what to do if they were tempted not to use sunscreen (Table 2).

**Table 2:** Sunscreen Use and Skin Self Examination (SSE) planning behaviour at baseline

		Planning behaviour									
		When		What		Where		How		Coping	
Health Behaviour		%	(*n*)	%	(*n*)	%	(*n*)	%	(*n*)	%	(*n*)
Sunscreen Use	1 – Completely disagree	23.3	(145)	28.5	(177)	30.5	(190)	34.6	(215)	39.8	(247)
	2	15	(93)	19.8	(123)	18.8	(117)	20.7	(129)	19.8	(123)
	3	23.5	(146)	23.8	(148)	17.4	(108)	20.9	(130)	19.8	(123)
	4	24.3	(151)	17.2	(107)	16.6	(103)	15.0	(93)	11.8	(73)
	5 – Completely agree	14.0	(87)	10.8	(67)	16.7	(104)	8.8	(55)	8.7	(54)
Skin Self Examination	1 – Completely disagree	68.8	(431)	–	–	64.8	(403)	65.1	(404)	63.3	(395)
	2	19.5	(122)	–	–	19.1	(119)	20.8	(129)	20.4	(127)
	3	8.6	(54)	–	–	10	(62)	10.3	(64)	13.1	(82)
	4	1.9	(12)	–	–	4.8	(30)	2.6	(16)	2.4	(15)
	5 – Completely agree	1.1	(7)	–	–	1.3	(8)	1.3	(8)	0.8	(5)

Adolescents who intended to use sunscreen had statistically significantly higher sunscreen planning behaviour for each of the five aspects of planning: *When* (*p* < 0.001); *What* (*p* < 0.001); *Where* (*p* < 0.001); *How* (*p* = 0.001); *Coping* (*p* = 0.001) (Table 3). For example, 86.2% (*n* = 150) of adolescents who intended to use sunscreen had made a plan *When* to use sunscreen compared to 40.5% (*n* = 121) who did not intend to use sunscreen (Table 3).

**Table 3:** Associations between sunscreen use and SSE and planning behaviour at baseline

	Planning Behaviour (% Yes)									
	When		What		Where		How		Coping	
Intention/behaviour	%	(*n*)	%	(*n*)	%	(*n*)	%	(*n*)	%	(*n*)
Sunscreen Use Intention										
Yes	86.2	(150)	79.8	(190)	75.4	(156)	81.1	(120)	82.7	(105)
Don’t know	62.2	(92)	54.8	(80)	61.1	(66)	66.9	(87)	68.3	(84)
No	40.5	(121)	39.2	(93)	46.1	(141)	45.5	(156)	46.9	(173)
Sig.	< 0.001		< 0.001		< 0.001		0.001		0.001	
SSE Intention										
Yes	9.4	(9)	–	–	11.6	(11)	11.7	(11)	9.4	(9)
Don’t know	3.5	(9)	–	–	7.0	(18)	4.3	(11)	0.8	(2)
No	0.4	(1)	–	–	3.4	(9)	0.4	(1)	3.5	(9)
Sig.	< 0.001		–	–	< 0.001		< 0.001		< 0.001	
SSE behaviour										
Yes	10.5	(4)	–	–	16.2	(6)	22.2	(8)	13.2	(5)
Don’t know	20.8	(5)	–	–	20.8	(5)	16.7	(4)	8.3	(2)
No	1.8	(10)	–	–	4.8	(27)	2.1	(12)	2.3	(13)
Sig.	< 0.001		–	–	< 0.001		< 0.001		0.001	

There were statistically significant associations between sunscreen use intention and CSM dimensions *identity* and *consequences.* Adolescents who intended to use sunscreen believed more strongly that skin cancer would affect their life compared to those who did not intend to use sunscreen (mean 8.33 versus 7.67, *p* < 0.001); adolescents who believed more strongly that skin cancer would be painful compared to those who did not intend to use sunscreen (mean 7.26 versus 6.53, *p* = 0.002). No statistically significant differences in sunscreen use intentions were observed for the CSM dimensions *control*, *timeline* (Table 4) *and causes* (Table 5).

**Table 4:** Associations between SSE behaviour, intention and CSM attributes at baseline

		SSE/SSE Intention/sunscreen use intention									Sig
		Yes^1^			Don’t know			No			
	CSM Attribute	n	Mean	SD	n	Mean	SD	n	Mean	SD	
Skin Self Examination (SSE)	*Identity:* How much skin cancer would affect your life?	38	7.66	2.122	561	8.13	1.888	24	8.13	2.071	0.375
	*Control:* How much control you feel you have to prevent skin cancer?	38	6.39	2.047	562	5.65	2.256	24	5.13	2.659	0.128
	*Timeline:* Given your age, how relevant to conduct SSE?	38	6.42	2.332	557	5.13	2.241	24	4.92	2.749	0.008**
	*Consequences*: How painful the effects of skin cancer would be?	38	7.24	2.307	557	7.02	2.014	24	6.75	2.625	0.707
SSE Intention	*Identity:* How much skin cancer would affect your life?	96	8.56	1.514	261	8.12	1.759	265	7.93	2.104	0.078
	*Control:* How much control you feel you have to prevent skin cancer?	96	6.00	2.088	261	5.98	2.057	265	5.24	2.433	< 0.001***
	*Timeline:* Given your age, how relevant to conduct SSE?	96	6.10	2.174	258	5.56	2.027	263	4.53	2.373	< 0.001***
	*Consequences*: How painful the effects of skin cancer would be?	95	7.40	1.991	260	7.04	2.000	263	6.89	2.125	0.101
Sunscreen Intention	*Identity:* How much skin cancer would affect your life?	363	8.33	1.749	156	7.81	1.947	100	7.67	2.265	< 0.001***
	*Control:* How much control you feel you have to prevent skin cancer?	363	5.80	2.129	157	5.60	2.287	100	5.36	2.607	0.190
	*Timeline:* Given your age, how relevant to conduct SSE?	359	5.29	2.214	156	5.19	2.246	100	4.95	2.560	0.412
	*Consequences*: How painful the effects of skin cancer would be?	362	7.26	1.916	154	6.79	2.016	100	6.53	2.460	0.002**

Note: ^1^ Aggregated from 5-point Likert scale where 1 = definitely will not and 5 = definitely will; 1–2 = No, 3 = Don’t know, 4,5 = Yes

**p < 0.01, ***p < 0.001

**Table 5:** Associations between CSM Causes (Age, Sex, Hereditary Factors) and SSE, intention and sunscreen intention at baseline

	Yes		Don’t know		No		Sig
	%	*n*	%	(*n*)	%	(*n*)	
Skin Self Examination (SSE)	9.8	19	5.2	10	85.1	165	0.006
SSE Intention	20.1	39	41.2	80	38.7	75	0.079
Sunscreen Intention	61.7	119	25.9	50	12.4	24	0.187

#### SSE behaviour, intention, planning and risk perceptions

At baseline, 6.1% (*n* = 38) of adolescents reported that they had examined their skin for signs of possible cancer in the past month and 90.1% (*n* = 563) had not (Table 5). No statistically significant gender differences were observed (Table 6).

**Table 6:** Skin Self Examination (SSE) behaviour at baseline by gender

	Total (*n* = 625)		Male (*n* = 342)		Female (*n* = 283)		Sig.
	%	(*n*)	%	(*n*)	%	(*n*)	
Yes	6.1	(38)	5.6	(19)	6.7	(19)	0.42
No	90.1	(563)	89.8	(307)	90.5	(256)	
Don’t Know	3.8	(24)	4.7	(16)	2.8	(8)	

4.6% (*n* = 29) of adolescents definitely intended to conduct SSE on a regular basis and 13.3% (*n* = 83) definitely did not intend to conduct SSE on a regular basis (Table 1). The difference between male and female intentions were statistically significant; more males definitely did not intend to conduct SSE than females (17% (*n* = 58) versus 8.8% (*n* = 25), *p* = 0.041) (Table 1).

1.1% (*n* = 7) of adolescents had made a detailed plan *When* to conduct SSE; 1.3% (n = 8) *Where* to conduct SSE; 1.3% (n = 8) *How* to conduct SSE; and 0.8% (n = 5) had made a *Coping plan* for dealing with things that could stop them from conducting SSE (Table 2).

Adolescents who had conducted SSE in the past month or intended to conduct SSE on a regular basis had statistically significantly higher SSE planning behaviour for each of the four aspects of planning: *When* (*p* < 0.001); *Where* (*p* < 0.001); *How* (*p* = 0.001); *Coping* (*p* = 0.001) (Table 3). For example, 10.5% (*n* = 4) of adolescents who had conducted SSE in the past month had made a plan *when* to conduct SSE compared to 1.8% (*n* = 10) who had not conducted SSE (Table 3).

There were statistically significant associations between SSE behaviour and CSM dimensions *timeline* and *cause.* Adolescents who had conducted SSE in the past month believed more strongly than those who had not conducted SSE in the past month that it was relevant to conduct SSE at their age (mean 6.42 vs. 4.92; *p* = 0.008) (Table 4). 9.8% (*n* = 19) of adolescents who did SSE in the previous month associated skin cancer *causes* with age, sex and constitutional factors and 4.3% (*n* = 17) who reported SSE in the previous month associated skin cancer causes with other factors (i.e. individual lifestyle factors or general environmental conditions) (*p* = 0.006) (Table 5).

There were statistically significant associations between SSE intention and CSM dimensions *timeline* and *control*. Adolescents who intended to conduct SSE on a regular basis believed more strongly than those who did not intend to conduct SSE that they had control to prevent skin cancer (mean 6.00 vs. 5.24; *p* < 0.001) and that it was relevant to conduct SSE at their age (mean 6.10 vs. 4.53; *p* < 0.001). No significant differences were observed for the CSM dimensions *identity, consequences* (Table 4) *and causes* (Table 5).

### Indicative intervention outcomes

#### Sunscreen use intention and planning

Comparisons between baseline and follow-up scores show that there were statistically significant beneficial changes in sunscreen use intention and sunscreen use planning behaviour for adolescents in the intervention group but not for adolescents in the control group (Table 7). For example, intention to use a high factor sunscreen significantly increased in the intervention group (mean 3.67 to 3.88, *p* < 0.001) but decreased in the control group (3.61 to 3.37, *p* = 0.015) (Table 7). However, after adjusting for age there were no statistically significant differences in sunscreen use intention and planning behaviour between baseline and follow-up in either the control or intervention groups (Table 7).

**Table 7:** Change in outcome measures between baseline and follow-up

	Control					Intervention				
	Baseline	Follow-up		Unadjusted	Adjusted	Baseline	Follow-up		Unadjusted	Adjusted
Outcome Measures	Mean (SD)	Mean (SD)	n	Sig	Sig	Mean (SD)	Mean (SD)	n	Sig	Sig
Sunscreen Use Intention and Planning Behaviour										
Intention to use sunscreen	3.61 (1.203)	3.37 (1.341)	79	0.015*	0.388	3.67 (1.177)	3.88 (1.099)	360	< 0.001***	0.186
Plan WHAT sunscreen intend to use	2.47 (1.440)	2.44 (1.366)	79	0.443	0.773	2.64 (1.353)	3.33 (1.268)	369	< 0.001***	0.261
Plan WHEN to use sunscreen	2.52 (1.376)	2.44 (1.366)	79	0.533	0.803	3.01 (1.378)	3.33 (1.268)	369	< 0.001***	0.817
Plan WHERE to get it	2.43 (1.420)	2.29 (1.360)	79	0.237	0.965	2.81 (1.479)	3.17 (1.358)	369	< 0.001***	0.779
Plan HOW to get it	2.27 (1.288)	2.10 (1.257)	79	0.198	0.952	2.52 (1.365)	2.93 (1.259)	368	< 0.001***	0.196
COPING Plan	2.11 (1.368)	2.04 (1.203)	79	0.513	0.479	2.32 (1.309)	2.91 (1.337)	366	< 0.001***	0.915
Skin Self Examination Intention and Planning Behaviour										
Intention to examine skin for possible signs of skin cancer on a regular basis	2.71 (0.865)	2.49 (0.932)	79	0.035*	0.456	2.62 (0.976)	3.04 0.978	375	< 0.001***	0.051
I have made a detailed plan regarding WHEN	1.47 (0.875)	1.49 (0.799)	79	0.300	0.125	1.48 (0.842)	2.21 (1.103)	376	< 0.001***	0.718
I have made a detailed plan regarding WHERE	1.56 (0.920)	1.56 (0.877)	78	0.991	0.569	1.61 (0.945)	2.62 (1.221)	374	< 0.001***	0.192
I have made a detailed plan regarding HOW	1.55 (0.935)	1.62 (0.901)	78	0.744	0.996	1.55 (0.858)	2.72 (1.213)	374	< 0.001***	0.022*
I have made a detailed plan for COPING	1.48 (0.830)	1.76 (0.950)	79	0.017*	0.672	1.65 (0.915)	2.37 (1.166)	372	< 0.001***	0.426
Skin cancer risk representations										
*Identity:* How much skin cancer would affect your life	7.70 (2.084)	7.71 (2.316)	77	0.729	0.888	8.22 (1.806)	8.26 (1.943)	375	0.215	0.474
*Control:* How much control you feel you have to prevent skin cancer	5.29 (2.251)	5.05 (2.197)	78	0.834	0.990	5.90 (2.254)	6.62 (1.893)	376	< 0.001***	0.029*
*Timeline:* Given you age, how relevant to conduct SSE	4.81 (2.391)	4.59 (2.265)	78	0.767	0.104	5.23 (2.308)	7.14 (2.068)	372	< 0.001***	0.873
*Consequences:* How painful the effects of skin cancer would be	6.74 (2.002)	6.21 (2.223)	76	0.053	0.869	7.05 (2.036)	6.96 (2.200)	369	0.524	< 0.001***
	% (n)	% (n)				% (n)	% (n)			
Skin Self Examination in past month										
(% Yes)	8.7 (8)	12.5 (13)		0.337(^+^)	0.599(^+^)	5.6 (30)	32.6 (143)		< 0.001*** (^+^)	< 0.001*** (^+^)
(% No)	85.9 (79)	82.7 (86)				90.8 (486)	65.1 (286)			
(% Don’t know)	5.4 (5)	4.8 (5)				3.6 (19)	2.3 (10)			
Talk about skin cancer in the past month										
(% Yes)	8.7 (8)	14.6 (15)		0.866(^+^)	0.888(^+^)	9.2 (49)	53.5 (234)		< 0.001***(^+^)	< 0.001***(^+^)
(% No)	91.3 (84)	85.4 (88)				90.8 (481)	46.5 (203)			

Note:*p < 0.05, ***p < 0.001

(+) *p*-values refer to the comparison between control and intervention for baseline and follow-up separately. Logistic regression model was applied to take into account confounder ‘Age’

#### SSE behaviour, intention and planning

Comparisons between baseline and follow-up scores show that proportionately more adolescents in the intervention group changed their SSE behaviour for the better compared with adolescents in the control group (5.6 to 32.6%, *p* < 0.001 vs. 8.7 to 12.5%, *p* = 0.337) (Table 7).

There was a statistically significant beneficial change in intentions to regularly conduct SSE in the intervention group (mean 2.62 to 3.04, *p* < 0.001) between baseline and follow-up whereas there was a statistically significant detrimental change in the control group (mean 2.71 to 2.49, *p* = 0.035) (Table 7). However, after adjusting for age no statistically significant differences in SSE intention between baseline and follow-up were observed in either the intervention (*p* = 0.051) or control (*p* = 0.035) groups (Table 7).

Comparisons between baseline and follow-up scores show that there was a significant beneficial change in SSE planning behaviour in the intervention group whereas there were no significant changes in the control group for planning *when, where* and *how* to conduct SSE (Table 6). For example, there was a significant beneficial change in planning *when* to conduct SSE in the intervention group (mean 1.48 to 2.21, *p* < 0.001) and no statistically significant change in the control group (mean 1.47 to 1.49, *p* = 0.3) (Table 6). There was a statistically significant beneficial change in *coping* planning in the intervention group (mean 1.65 to 2.37, *p* < 0.001) and in the control group (mean 1.48 to 1.76, *p* = 0.017) (Table 7). After adjusting for age no statistically significant changes were observed for any of the four measures of SSE planning in the control group, and the only measure that remained statistically significant in the intervention group was planning *how* to conduct SSE (*p* = 0.022) (Table 7).

#### Talk about skin cancer

At baseline, 9.2% (*n* = 57) of adolescents reported that they had talked to someone in the past month about skin cancer (Table 1). No statistically significant gender differences were observed (Table 1). The number of adolescents talking about skin cancer in the last month that received the intervention significantly increased after the intervention (9.2 to 53.5%, *p* < 0.001); no statistically significant increase was observed in the control school (8.7 to 14.6%, *p* = 0.866) (Table 7).

#### Skin cancer risk representations

Comparisons between baseline and follow-up scores show that there were no statistically significant changes in CSM dimensions in the control group (Table 7). In the intervention group, before adjusting for age, there were statistically significant changes in CSM dimensions *control* and *timeline*; adolescents in the intervention group believed more strongly that they had control to prevent skin cancer at follow-up than they did at baseline (mean 5.90 versus 6.62, p < 0.001) and believed more strongly that it was relevant to conduct SSE at their age (7.14 versus 5.23, p < 0.001) (Table 7). After adjusting for age, changes in the CSM dimension *control* remained statistically significant (*p* = 0.029) and change in the *consequences* measure was statistically significant (*p* < 0.001) (Table 7).

## Discussion

A key purpose of conducting this feasibility study was to evaluate trial parameters. The response rate from schools was low (only 5 out of 37 schools that were approached agreed to participate) and reflects busy curricula timetables at the time of year when students aged 15–16 years in the UK are studying for exams. Our cancer awareness intervention trials conducted with younger adolescents who are not in an examination period have experienced a much higher school response rate [62]. Thus, the lead up to examination revision and examinations should be avoided in future research with this age group. Nonetheless, no parent/carer opted their child from the study and no student declined to participate, suggesting that it was an acceptable intervention. The proportion of students on the school register completing baseline and follow-up questionnaires was 50.5%, which we believe could be improved if the study was conducted outside of the examination revision period. The proportion of students in intervention schools receiving the intervention was high (81%) but only a third of students completed the home-based assignment. Our findings suggest that the number of students completing the home-based assignment could be improved if adolescents saw the relevance of SSE (a key part of the assignment) for their age group.

The onset of multiple risk behaviours, including excessive sun exposure, cluster in adolescence and there is a recognised need to determine the effectiveness of interventions to address this problem [23]. Hence, a further purpose of the feasibility study was to determine if the intervention worked as intended. The study shows that at baseline, 58.5% of adolescents intended to use a high factor sunscreen if they were going out in the sun and that female adolescents had a greater intention to use sunscreen than males. This finding is similar to other studies conducted in the UK [16, 63] and elsewhere [17, 64–66]. The number of adolescents who reported that they had conducted SSE was low (6.1%) and only 15.3% intended to examine their skin on a regular basis. This is similar to a figure of 4% of 16 to 25 year olds reported by a survey conducted in Northern Ireland [40]. We found females had a greater intention to conduct SSE on a regular basis than males. Other studies have also found that SSE is associated with being female [35]. Taken together, this body of work provides convincing evidence of the need for interventions to increase sunscreen use and SSE in this age group. The feasibility study was not designed to definitely measure effectiveness but rather to give an indication that the intervention worked as intended. As hypothesised, the study suggests that the intervention will facilitate beneficial changes in adolescent sunscreen use intention and SSE behaviour and intention, and adds to the body of work reporting the beneficial effects of psycho-educational interventions on sun protection behaviours in adolescents [48, 66–70]. There is, however, only a very limited body of work about SSE in adolescents to which we can compare our findings. A study of Turkish teenagers (mean age 13 years) found significant increases in intentions to conduct SSE following an educational intervention [71]; however, the study did not include a control group. The feasibility study highlights that the use of personal stories is an important method for delivering crucial health messages. In this intervention, personal stories presented by video (‘Dear 16 year old me’) or in-person (young adult cancer survivor) were remembered by adolescents 8 weeks after the intervention was delivered in schools. Our qualitative findings also suggest that a skin cancer presentation delivered in schools will provide further potential teachable moments between parents/carers and their child about sun protection.

Understanding the pathways through which behaviour change occurs is important during feasibility work; a key purpose of this feasibility study was to determine if the above observed intervention effects on sunscreen use intention and SSE behaviour/intention occurred through hypothesised pathways of theoretical mediation. To evaluate this, we first examined associations between planning, illness perceptions and sunscreen use and SSE at baseline and second, we measured changes in planning and illness perceptions from baseline to follow-up. As hypothesised, at baseline, we found significant associations between planning and sunscreen use intention, SSE behaviour and SSE intention. Other studies have also reported associations between planning and sunscreen use in adolescents [18] and adults [72] and between planning and SSE [43]. A mobile text messaging-delivered behavioural intervention in a population aged 18 to 42 years found that those who had made plans to check their skin for early signs of skin cancer were more likely to conduct SSE [43]. There is therefore a growing body of empirical evidence to corroborate theoretical models suggesting that behavioural intentions are more likely to be translated into action when people generate specific plans [45, 56] and points to the inclusion of planning activities in interventions to increase sunscreen use and SSE in adolescents. We also, as hypothesised, observed significant associations at baseline between illness perceptions (including risk perception) and sunscreen use intention and SSE behaviour/intention. Hence, our study findings are consistent with other research showing associations between risk perceptions and sunscreen use in adolescents [18, 21, 48, 49, 73] and between the CSM dimension *timeline* and SSE in young adults [54] and are consistent with the thesis that risk perceptions influence behaviour [53]. We can only speculate reasons why we did not consistently find associations between all five CSM dimensions and sunscreen and SSE behaviour/intention. It could be that some illness perceptions are more influential than others and vary by behaviours. However, a more likely explanation is that our findings are an artefact of study design; perhaps it is not surprising that we did not observe an association between relevance beliefs (i.e. measured in this study by the CSM dimension *timeline*) and sunscreen use intention because we only included one item and that item related specifically to adolescents’ perceived relevance of SSE during adolescence and we did not measure their perceived relevance of using sunscreen during adolescence. Importantly, we only operationalised illness perceptions using CSM dimensions of illness representation. It has been suggested that different risk perception operationalisations explain the inconsistent findings in literature regarding the relationship between risk perceptions and cancer-related behaviours [74]. Hence, future studies should consider operationalising illness perceptions using a range of behaviour change theories and theoretical constructs.

### Limitations

Our study provides new evidence regarding interventions that simultaneously address sunscreen use to prevent skin cancer and SSE to improve early detection that was previously lacking internationally. However, several limitations of the study must be noted. First, the sample consists of a small number of mainly white British adolescents selected from only five schools and so may not generalise to populations with different cultural backgrounds. Second, the study was conducted during January to March when it is cold in the UK and when the population is less likely to think about use of sun protection. Whether the findings would be similar during the summer months is unclear but given that we found the intervention appeared to influence sunscreen use and SSE when the population is possibly less receptive to sun safe messaging we are confident that this would be the case. Third, the study relied on self-report and is therefore prone to social desirability reporting biases. In this study, we did not measure actual sunscreen use (we only measured intention) nor did we examine associations between sunscreen use and sunburn incidence. If sunscreen use does not lead to a reduction in sunburn then even if an intervention were to improve sunscreen use it would not necessarily improve health outcomes unless sunburn declined as a consequence of increasing sunscreen use. Further research should therefore consider objective measurement of sunscreen use and sunburn incidence. Similarly, any study that relies on self-reported SSE is subject to recall bias, and may lead to the overestimation of SSE behaviour. Increasing use of mobile photo-documentation means that future studies may be in a position to include objective measurement of SSE [75, 76]. We did not ask participants whether they examined their whole body during SSE, only arms and legs, and how SSE is measured can yield different results [35]. Fourth, as mentioned previously, risk perception was only operationalised using CSM and other behaviour theories could strengthen the intervention. This is because there are other social cognitions that may influence behaviour and should therefore be considered in future studies. For example, outcome expectancy (a belief about the likelihood of the behaviour leading to a *specific* outcome) has been associated with sun protection and SSE [43] and appearance motives have been associated with sunscreen use intention in adolescents [67]. Fifth, while promising, our study suggests that this brief intervention on its own has limitations. Our qualitative findings suggest that adolescents may defer using sunscreen because they do not like its texture or smell. Whether the intervention messages about sun protection are sufficiently powerful to overcome these barriers in the long-term is unclear from this study. Adolescents also questioned the relevancy of SSE during adolescence. While they understood and retained the message that sunburn in childhood and adolescence increases their chances of skin cancer, they also understood and retained the message that the negative effects on health are likely only to be apparent many years later. Making SSE habitual by beginning in adolescence may therefore prove challenging. We may need to develop *‘Do you know your skin?’* by increasing intervention intensity, the relevance of SSE during adolescence, and consider combining it with other interventions (e.g. increasing availability of adolescent-friendly sunscreen) as part of a larger programme of effort to promote sunscreen use and SSE.

## Conclusion

This study suggests that it is feasible to simultaneously promote sun safe behaviours and skin self-examination using a theory-based psycho-educational intervention but further research is required to improve study uptake, intervention adherence and effectiveness.

## Additional files

Additional file 1: CONSORT flowchart for trials. Number of participants screened, enrolled, allocated to intervention or control arms, and follow up and assessment rates. (DOCX 64 kb)

Additional file 2: Qualitative data from the focus groups. Table with thematic headings and related quotations from participants. (DOCX 102 kb)

## Abbreviations

CSM: Common-sense model of illness representation
SSE: Skin self-examination
