# Supplementary material for: Promoting sunscreen use and skin self-examination to improve early detection and prevent skin cancer: quasi-experimental trial of an adolescent psycho-educational intervention
Source: BMC Public Health. 2018 May 29;18:666. doi: 10.1186/s12889-018-5570-y (PMC5975556; doi:10.1186/s12889-018-5570-y)
Supplement: Supplementary file 1 — CONSORT flowchart for trials. Number of participants screened, enrolled, allocated to intervention or control arms, and follow up and assessment rates. (DOCX 64 kb) [file 12889_2018_5570_MOESM1_ESM.docx]

Assessed for eligibility (students n=919)

Excluded (students n=0)

Assessed at baseline (students n= 535)

Assessed at follow up (students n=439)

Lost to follow-up (give reasons) (schools n=0; students n=0)

Allocated to intervention (schools n= 4; students n= 771)

♦ Received allocated intervention (students n=639)

♦ Did not receive allocated intervention (give reasons) (n=132 due to absence from school)

Allocated to control (schools n=1; students n=130)

## Allocation

## Assessment

## Follow-Up

## Enrollment

Screened prior to eligibility assessment (5 schools (clusters) students n= 901)

Excluded (students n=0)

## Screened

Assessed at baseline (students n=92)

Assessed at follow up (students n=104)

Lost to follow-up (give reasons) (students n=0)

Lost to follow-up (give reasons) (students n=0)
