# Supplementary material for: Promoting sunscreen use and skin self-examination to improve early detection and prevent skin cancer: quasi-experimental trial of an adolescent psycho-educational intervention
Source: BMC Public Health. 2018 May 29;18:666. doi: 10.1186/s12889-018-5570-y (PMC5975556; doi:10.1186/s12889-018-5570-y)
Supplement: Supplementary file 2 — Qualitative data from the focus groups. Table with thematic headings and related quotations from participants. (DOCX 102 kb) [file 12889_2018_5570_MOESM2_ESM.docx]

***Supplementary File: Focus group themes***

| ***Theme*** | ***Quotations*** |
| --- | --- |
| *Personal experiences of cancer* | *Participant:* Whereas like the actual presentation was a bit boring and then he [the young adult cancer survivor who gave the brief talk] was like, he like, it was more easy to relate to him because he was like young and stuff I guess.  *Participant:* He [the young adult cancer survivor who gave the brief talk] probably would have been about our age and not had much knowledge about it [skin cancer] and how it affected him in quite a major way, and even now he still has to cover himself in sun cream and all that is more about the effect.  *…*  *Researcher:* Can you remember what sort of things he said?  *Participant:* He said that he originally thought it was like a spot and then his mum and dad kinda like, they ignored it, but no, sorry, *he* ignored it but his mum got creams and stuff and took him to the doctors, the first time they said it was fine, and then it was like the second time they sent him to a specialist (School 3).  *Participant:* I liked the, from like the younger guy who’d experienced it [skin cancer] himself, that was the best bit. I think that was the most impactful bit, because he was just like a normal guy, only a little older than us, I think that was like stuck in your memory most.  *…*  *Participant:* I quite liked the video because it didn’t just give like one perspective…  *Participant 3:* They make it real, like someone is actually telling you that they've actually had it. It puts it [skin cancer] into real life, that it can actually happen to someone. What you're saying it's hypothetical whereas, so you're telling us this would happen, but if someone tells us who’s had it it's a more personal reflection on what happens and therefore you can connect more in my opinion. (School 1)  *Participant:* I feel it tried a bit too hard to appeal to young people. It felt very teenager‑focused, a bit direct and dramatized. I don't know how you’d say it, directed towards young people, a bit patronising. I mean they make videos with a special purpose and you could tell that they tried quite hard to be ‘down with the kids’ or whatever. I think that just turns people off, like if it was just like a genuine video of people talking that would be better than it's all like audio and editing that's meant to be ‘cool’ and interesting for us to watch. It made an impact but I think it is crazy, for example, the music in the background. (School 1) |
| *Distaste for sunscreen* | *Investigator:* Have you had sunburn before?  *Participant:* At the back where I'd forgotten [to put sunscreen on] … it got really sunburnt quite bad, and it peeled a bit and everything. And I hadn’t had anything like that before. So, after the presentation I was quite worried. The whole thing about, like one bad sunburn can double your chances. (School 1)  *Researcher:* How do you feel about wearing sun cream?  *Participant:* That stuff stinks though.  *Participant:* I know it's good, I don’t get burnt. (School 3)  *Participant*: I hate the texture of sun cream.  *Participant:* It's horrible.  *…*  *Participant:* So, you know how you get a dry shampoo? It should be like that with sun cream so it’s just not greasy.  *Participant:* And it sticks to your clothes as well.  *Participant :* I hate how it just like lies on your skin, you just kind of feel a bit ‘minging’ lying there. (School 1). |
| *Relevance of SSE in adolescence* | *Participant:* I just think what I took away from it was like that you need to check yourself for these (e.g., moles) quite regularly. (School 1)  *Researcher*: Did you do the home assignment, the skin examination on the booklet?  *Participant:* I had a wee look because I have a lot of freckles and stuff, but not like marking it out on the sheet like it [the booklet] said to. (School 1)  *Participant:* I knew that it asks us routinely check it, but if I haven't got it in 14 years then, I assume that like in the next few years I will get it so like routinely checking it once a week wasn't really going to make that much difference. (School 3)  *Researcher*: How are we going to get you to do the homework?  *Participant:* Tell us like when it has to be done by? (School 2) |
| *Skin cancer conversations* | *Participant:* I spoke to my mum just a little bit about it, just after school, and she’s already really, really paranoid about sunscreen, so she was like trying to put it on ten minutes when there's a tiny bit of sun… So, I wasn't even going to mention to her that we’d had a talk because I knew she would just be “I told you all the dangers about what can happen when you don’t wear sun cream and stuff …”  *…*  *Participant:* Em, I just spoke to my, like my mum about it like when I was at home and we were just kinda like talking really kind of, em the, we were just like talking about like different types of like cancers as well, and we were just like talking about how like on holiday like you do have to like wear the sun creams. (School 1)  *Participant:* I just left the booklet out on the kitchen table …then she looked at it and that’s it.  *…*  *Participant:* I just mentioned it at the dinner table. (School 2) |
